# No-boundary thinking in bioinformatics research

**DOI:** 10.1186/1756-0381-6-19

**Published:** 2013-11-06

**Authors:** Xiuzhen Huang, Barry Bruce, Alison Buchan, Clare Bates Congdon, Carole L Cramer, Steven F Jennings, Hongmei Jiang, Zenglu Li, Gail McClure, Rick McMullen, Jason H Moore, Bindu Nanduri, Joan Peckham, Andy Perkins, Shawn W Polson, Bhanu Rekepalli, Saeed Salem, Jennifer Specker, Donald Wunsch, Donghai Xiong, Shuzhong Zhang, Zhongming Zhao

**Affiliations:** 1 Department of Computer Science, Arkansas State University, Jonesboro, AR 72467, USA; 2 Sustainable Energy & Education Research Center, University of Tennessee at Knoxville, Knoxville, TN 37996, USA; 3 Department of Microbiology, University of Tennessee, Knoxville, TN 37996, USA; 4 Department of Computer Science, University of Southern Maine, Portland, ME 04104, USA; 5 Arkansas Biosciences Institute, Department of Biological Sciences, Arkansas State University, Jonesboro, AR 72467, USA; 6 Department of Information Science, University of Arkansas at Little Rock, Little Rock, AR 72204, USA; 7 Department of Statistics, Northwestern University, Evanston, IL 60208, USA; 8 Center for Applied Genetic Technologies, The University of Georgia, Athens, GA 30602, USA; 9 Arkansas Science & Technology Authority, Arkansas NSF EPSCoR, Little Rock, AR 72201, USA; 10 Arkansas High Performance Computing Center, University of Arkansas at Fayetteville, Fayetteville, AR 72701, USA; 11 The Geisel School of Medicine, Dartmouth College, Lebanon, NH 03756, USA; 12 Department of Basic Sciences, College of Veterinary Medicine, Mississippi State University, Jackson, MS 39762, USA; 13 Department of Computer Science and Statistics, University of Rhode Island, Kingston, RI 02881, USA; 14 Department of Computer Science and Engineering, Mississippi State University, Jackson, MS 39762, USA; 15 Center for Bioinformatics and Computational Biology, University of Delaware, Newark, DE 19711, USA; 16 National Institute for Computational Sciences, Department of Electrical Engineering and Computer Science, UTK and ORNL, Oak Ridge, TN 37832, USA; 17 Department of Computer Science, North Dakota State University, Fargo, ND 58102, USA; 18 Graduate School of Oceanography, University of Rhode Island, Narragansett, RI 02882, USA; 19 Department of Electrical & Computer Engineering, Missouri University of Science & Technology, Rolla, MO 65409, USA; 20 Department of Pharmacology and Toxicology, Medical College of Wisconsin, Milwaukee, WI 53223, USA; 21 Department of Industrial and Systems Engineering, University of Minnesota, Minneapolis, MN 55455, USA; 22 Department of Biomedical Informatics, Vanderbilt University School of Medicine, Nashville, TN 37203, USA

**Keywords:** No-boundary thinking, Human infrastructure

## Abstract

Currently there are definitions from many agencies and research societies defining “bioinformatics” as deriving knowledge from computational analysis of large volumes of biological and biomedical data. *Should this be the bioinformatics research focus?* We will discuss this issue in this review article. We would like to promote the idea of supporting human-infrastructure (HI) with no-boundary thinking (NT) in bioinformatics (HINT).

## The Big-data paradigm

Today’s data-intensive computing (“big data”) was advocated by the 1998 Turing Award^1^ recipient, Jim Gray, as the fourth paradigm for scientific discovery [1] after direct experimentation, theoretical mathematical physics and chemistry, and computer simulation.

With the rapid advance of biotechnologies and development of clinical record systems, we have witnessed an exponential growth of data ranging from “omics” (such as genomics, proteomics, metabolomics, and pharmacogenomics), imaging data, to electronic medical record data. Last year, the federal funding agencies National Institutes of Health (NIH) and National Science Foundation (NSF) exercised a joint effort to launch big-data initiatives and consortia to promote and support big-data projects [2]. Focused specifically on computational medicine and personalized treatments, large consortia have been initiated (such as The Cancer Genome Atlas (TCGA); http://cancergenome.nih.gov/) to collect large quantities of data and conduct analyses with the hope of addressing cancer causes, diagnosis, prognosis, and treatments.

Big-data helps drive knowledge discovery and brings opportunities to research; however, significant science challenges remain. Big-data has its problems and dilemmas; *we think we should discuss and re-think about it.*

The **NSF EPSCoR Workshop in Bioinformatics to Foster Collaborative Research**, which was held in Little Rock during March 3–5, 2013, attracted attendants from approximately thirty states. Attendees included faculty, research scientists, technical staff, and students in the areas of computer science, mathematics, statistics, engineering, biology, biochemistry, biophysics, and biomedical sciences. A new scientific thinking was presented and discussed at the workshop: **No-boundary thinking in bioinformatics research**.

### The scientific perspective

Ultimately, the goal of research is to address scientific challenges. However, arguably we have lost track of this goal by focusing too heavily on collecting and analyzing “big data.” Should the real challenges in bioinformatics be driven by big-data or by science? Of course, we think the latter is of paramount importance:

***“Einsteins in bioinformatics”.*** *When Einstein formulated his most significant intellectual contributions, was he working on the problems defined in math, in physics, or in philosophy? Were the problems solved by his knowledge in math, in physics, or in philosophy? While certainly well-versed in a variety of disciplines, he nurtured his ability to think outside the box that had limited the other scientists and researchers. This century needs “Einsteins in bioinformatics”, who are driven by the nature of the science problem but not its derivatives, whose approaches are not limited by disciplines, traditions, vocabularies, or even technologies. In short, “Einsteins in bioinformatics” approach science challenges with no discipline-boundary thinking.*

1 Defining the science problem is the most important. Einstein said, “If I had an hour to solve a problem, I’d spend 55 minutes thinking about the problem and 5 minutes thinking about solutions.”

2 **Defining science problems with no-boundary thinking.** A well-defined, real-world science problem should be based on knowledge of a variety of disciplines, but not from a specific currently-defined discipline or several disciplines. We need to define the science problem with no-boundary thinking, without boundaries of disciplines.

Our current practices do not effectively incorporate no-boundary thinking. Here is a general workflow of the **current strategy** to address science challenges in bioinformatics:

1 Wet labs gather experimental data and prepare samples.

2 Current “omics” technologies collect large amount of data.

3 Existing or new computational/mathematical/statistical methods are applied.

4 Results from the computational analysis are then validated by further wet-lab testing.

5 If needed, this process of 1–4 is repeated with refinements or expansions.

However, *it is important to have interdisciplinary thinking, no-boundary thinking, at the beginning* (even before designing biological and biomedical experiments, before preparing samples, before starting large data collection), not after getting back the collected data. According to the current workflow, after data collection through wet labs or advanced techniques, what is left for the next step is just technical analysis. Sometimes the collected data may not even be statistically meaningful and it is too late to fix it. Here bioinformatics research needs real computational thinking, real prediction modeling, and real interdisciplinary thinking at the beginning. Research, including bioinformatics research, ought to be science-driven, instead of data-driven.

It is becoming more and more clear that big-data is not equal to knowledge and is far from addressing scientific challenges. At the NSF bioinformatics workshop, the scientific community is re-thinking/questioning the gains of the big “omics” data and the Human Genome Project and also the other related international projects and initiatives (e.g., the 1000 genomes project, the HapMap Project). The fundamental contributions of these projects and the data they developed were clearly recognized as bringing significant value to the field. However, in spite of early promises linking “omics” data to a windfall of new cures and transformative discoveries, the conversion of knowledge to discovery and application has remained limited and challenging. Also there were insightful thinking and discussions at the next-generation sequencing (NGS) session panel discussion.

*It is widely known from 1900 to 1903, Marie and Pierre Curie, from several tons of the original material pitchblende, isolated one decigram of almost pure radium chloride and determined radium’s atomic weight as 225. They determined to isolate the new element from* tons of pitchblende*, because Marie had gone through the whole periodic system and believed that the substance they extracted contained a metal never known before. Marie had the absolutely revolutionary discovery: the ability to radiate did not depend on the arrangement of the atoms in a molecule, it must be linked to the interior of the atom itself. From a conceptual point of view it is her most important contribution to the development of physics (*http://www.nobelprize.org/nobel_prizes/themes/physics/curie/*).*

Is gathering more data like getting more pitchblende? When do we focus on the “radium extraction?” With more and more data, are we closer to the scientific goal of “getting radium extracted”, or we are just being indulged in the big-data? It is the time for us to re-think and clearly define the scientific goal, to work directly towards to the scientific goal!

### The bioinformatics perspective

The current situation in bioinformatics research reflects a two-sided problem: On one side, experts and researchers in math and computer science can be intimidated by the complexity of life sciences and the inability to provide precise solutions to life science problems. They tend to think bioinformatics is just applications of math and computational science. Math and computational scientists in bioinformatics do need to make the effort to develop very good understanding of biology and biomedical sciences. On the other side, there might be over-emphasis on hypothesis testing of wet labs, and we tend to think science challenges are challenges for life scientists and biomedical researchers, who now are getting big-data and just need the help from the math and computational side. Projects/consortia with more focus on life sciences and biomedical science are getting big bioinformatics resources and funds; many of those big labs simply hire and train math or computer science personnel as post-doctoral fellows or technicians to perform data analyses. *We can see that both the life science side and the computational side will not gain significantly. In the long run this kind of situation is not healthy for the development of bioinformatics research.*

The lack of full intellectual integration not only limits optimal development of bioinformatics research and life science areas, but also limits the development of the computational side. Even if one’s theoretical algorithm is later adapted and applied to solve a science problem and provide a profound input, you may not share in that success. You won’t feel that level of pride, since you are not directly linked with the outcome. We know big needs in science will always help push forward big “bursts” or “break-throughs” in mathematics, even in theoretical mathematics.

Let us think about a new situation: both computational and life science sides vested in the science project: co-developing the problem, co-solving the problem, both having ownership of the outcome. They will work hard and communicate a lot; they will feel this is “their” project. This situation is very different from the current situation when researchers from one side think that their work is like those of technicians. Science challenges calls for researchers from both sides to have the motivation, enthusiasm and creativity that are needed to make a difference.

There are some barriers, of course, such as academic structures, discipline-specific terminologies, and especially the mind-set. As researchers, we need to make efforts, e.g., to learn the terminologies, to open our minds and to move out of our intellectual comfort zones. *We would like to help build a bioinformatics research community that appreciates this sense of diversity, values this level of collaboration, and promotes this kind of mindset.*

**Bioinformatics is not just a middle-ware**, with experimental data collection at one end and verification of computational analysis results at the other end. Bioinformatics research is not at all just software “black-boxes” between computational scientists and life scientists! Bioinformatics needs researchers to understand classical and current approaches, to apply and effectively use them to empower biological and biomedical discoveries. Bioinformatics research also needs “human infrastructure” working at the interface, thinking at the interface, in order to address biological and biomedical challenges.

### Human infrastructure (HI) support

To better address real science challenges, **we as a group of scientists from different disciplines promote the idea of supporting human infrastructure with no-boundary thinking in bioinformatics.**

Bioinformatics research resources broadly include:

• hardware (locally- or remotely- accessible computers, servers and network broadband, high-performance computing (HPC), cloud computing),

• software and data (commercial or publicly-available software tools; data: -omics data, medical, molecular and clinical data, etc.), and

• wet-lab resources (sequencers, mass spectrometers, cell sorters, etc.) for wet-lab experiments and verification.

While big data, hardware, software, and wet-lab resources are important, they are not the most crucial for bioinformatics research. We think the most crucial need for current bioinformatics research is human infrastructure resource.

• We need to support researchers – human-infrastructure – to address science challenges with no-boundary thinking, to define science problems without the bound of disciplines - not just researchers who develop theoretical approaches or application tools for bioinformatics, not just researchers who apply statistical and computational tools to help life scientists.

• We need to support researchers – human-infrastructure – to work on **computational and mathematical modeling as the basis for science problem-solving**: Bioinformatics research is related to a broad spectrum of disciplines such as mathematics, computer science, statistics, biology, biochemistry, biophysics, bioengineering, and biomedicine – but the basis of bioinformatics research is modeling (computational and mathematical modeling). The last century saw how much mathematics impacted the development of fields such as chemistry and physics. This century, mathematics is greatly affecting the field of bioinformatics: Bioinformatics problem-solving needs design models - new biologically-meaningful models, and needs develop approaches - novel effective and efficient approaches. *Mathematics will revolutionize bioinformatics in this century just as mathematics revolutionized chemistry and physics in the last century.* Universities and institutions are becoming more aware that bioinformatics needs understanding of both the math/computation and life sciences sides. Many academic programs are being built, such as biomathematics, mathematical biology, or computational medicine, to help train/grow researchers with understanding of the related fields, and working at the frontier, working at the interface, thinking at the interface.

History shows us that great achievements and significant scientific research results usually come from researchers with passion for new knowledge who think outside the established paradigms or the predefined disciplines. The Curies did their work in an abandoned shed (Refer to: http://www.aip.org/history/curie/resbr2.htm for the picture of the “miserable old shed” where radium was isolated) and hired their first lab assistant only after collecting their Nobel Prize money. We would like to have proper ways to support our brilliant researchers.

With the research budget reductions of government agencies like NSF and NIH, should we re-think about how to effectively support science projects and how to reorient science directions? Instead of a continuous push to support big projects and big data, maybe we should consider a more effective way of addressing real science challenges. We think supporting human infrastructure (HI), “a distributive HI network”, could help mitigate the negative impacts of the current budget reductions. With bioinformatics as an inter-disciplinary research area and in the high-speed development stage, it might not be easy for current traditionally-organized departments to support the bioinformatics researchers and build the needed research positions. For scientific studies, interdisciplinary/multi-disciplinary research is becoming the norm. To help address the current need of the bioinformatics research field, universities should “borrow” this multi-disciplinary idea – collaborate with other universities to build research positions that collectively build the crucial HI capacity. If done strategically, there is the potential to save money and reduce redundancy and resource waste, while creating a more powerful and innovative infrastructure supporting bioinformatics.

### Summary

From our discussions, we think bioinformatics research needs support for human-infrastructure (HI) with no-boundary thinking (HINT) to address the challenging scientific problems. We need to nurture “Einsteins in bioinformatics”; we need to plant the seeds of “big” researchers (not more “big data”). These are the researchers who will push for breakthroughs in bioinformatics with no-boundary problem definition and problem solving.

## Endnotes

^1^The Turing Award is considered as the Nobel Prize in computer science.

## Competing interests

The authors declare that they have no competing interests.

## Authors’ contributions

XH conceives and brings up the idea of No-boundary thinking in bioinformatics, and drafts the manuscript; all the authors have been involved in discussing and help shaping the idea of No-boundary thinking, drafting or revising the manuscript, and have given approval for publication.
